# Hot topics at the intersection of aging and energetics: Diabetes/insulin resistance, Sirtuins, and the Microbiome

**DOI:** 10.12688/f1000research.5625.1

**Published:** 2014-10-28

**Authors:** Dudley W. Lamming

**Affiliations:** 1 Division of Endocrinology, Department of Medicine, University of Wisconsin-Madison and William S. Middleton Memorial Veterans Hospital, Madison, USA

## Abstract

A recent review in *F1000Research* identified the “top research priorities identified in leading publications” at the interface of Aging and Energetics. The authors identified the ten most-cited papers in each of the years 2010 through 2013, and used these forty papers to identify thematic categories. However, the search methodology used by the authors omitted many high-impact aging manuscripts. Minor modifications in the authors’ search methodology finds that Diabetes/insulin resistance, Sirtuins, and the Microbiome are also top thematic categories.

## Correspondence

My laboratory members and I were excited to read a recent review in *F1000Research* ^1^, entitled “Aging and energetics’ ‘Top 40’ future research opportunities 2010–2013”. The review discusses research opportunities in 10 fields at the intersection of aging and metabolism. The authors identified ten research categories through a Scopus search for the ten most-cited papers in each of the years 2010 through 2013, for a total of 40 papers. However, upon examining the list of these papers, I was surprised to notice that a significant number of these articles were reviews, and that I recognized very few of the papers. I was also struck by the relatively low number of citations each paper had gathered, as well as by the almost complete absence of high-impact journals such as *Cell*, *Nature*, *Science* and *PNAS* from the search results.

Helpfully, the authors included their Scopus search terms in the methods section of their review, and I was easily able to replicate the results of their search. Upon consideration of the search terminology, I realized the authors had limited their results to papers in which “aging”, “lifespan” and other key search terms relating to aging and metabolism appeared in the title. I repeated their search with one important difference: I searched for keywords and did not limit my search to the title field (see Methods). My list of the 10 most cited articles from each year (2010–2013) at the intersection of aging and metabolism/energetics is given in Appendix A.

The list I generated is almost entirely different from the authors’ results, including only 3 of the 40 papers identified by Allison and colleagues. My search identified many more high impact papers (based on citation count) and a greater proportion, 18 out of 41 papers, were published by the high impact journals *Cell*, *Nature*, *Science* and *PNAS*. Whereas Allison *et al.* identified Morselli *et al.* ^2^ as the original research manuscript in 2010 with the most citations (99), my methodology identified 8 papers from 2010 with a higher number of citations, the highest of which, Ng *et al.* ^3^, had 298 citations.

I performed a rapid scoring of my resulting list against the thematic categories identified by Allison and colleagues. Interestingly, “nutrient effects beyond energy” rose to the top of this list, due in part to my inclusion of metformin-related papers in this category. A new thematic area, Diabetes/insulin resistance, was tied for second place with Mitochondria, reactive oxygen species, and cellular energetics. This was followed by another new thematic area, “Sirtuins”, tied with “Calorie restriction”. The Microbiome was also an important new thematic area. Three categories identified by Allison *et al.*, “use and effects of mesenchymal stem cells (MSCs)”, “accretion and effects of body fat”, and “the aging heart”, were entirely absent from my newly generated list.

In conclusion, a minor change in search methodology resulted in the retrieval of a very different list of papers than that identified by Alison and colleagues. “Calorie restriction”, “nutrient effects beyond energy”, “mTOR”, and “autophagy” are still clearly important areas for aging research. However, Diabetes/insulin resistance, Microbiome, and Sirtuins should be included in this list. The narrow search strategy employed by Allison *et al.* missed these important thematic areas.

## Methods

I repeated the Scopus search the authors ^1^ had performed, substituting the use of the “KEY” (keyword) field in place of the “TITLE” (title) field. The search performed was:

( ( KEY ( ( aging OR ageing OR lifespan OR longevity OR senescence ) ) AND PUBYEAR > 2009 AND PUBYEAR < 2014 ) AND ( KEY ( ( calori* OR diet* OR energetic* OR nutri* OR food OR fat OR adipo* OR "body composition" ) ) AND PUBYEAR > 2009 AND PUBYEAR < 2014 ) )

For each search year (2010–2013), I then excluded review articles and removed self-citations using the Scopus tools, and then manually removed additional review articles and articles that did not involve biological research in the field of aging (i.e., consensus treatment guidelines, articles on children in developing countries, and material science manuscripts). Eleven manuscripts were included in 2012 due to a 10 ^th^ place tie in citation count.

Thematic areas were annotated by inspection of the title and abstract only. Themes were taken from the list generated by Allison *et al.*, and the following areas were added after inspection of the titles only: Diabetes/insulin resistance, Microbiome, and Sirtuins. Proteostasis was added to the Autophagy category, and Metformin was added to the “Nutrient effects beyond energy” category during the scoring process.

## Supplementary materials

**Appendix A: Top ten most cited papers on aging and energetics using keyword field for each year between 2010 and 2013.**

An alternative top 40 list to that generated by Allison *et al.* ^4^ using the KEY field in Scopus instead of the TITLE field.

Click here to access the data.

http://dx.doi.org/10.5256/f1000research.5625.s37562
