# Supplementary material for: Hot topics at the intersection of aging and energetics: Diabetes/insulin resistance, Sirtuins, and the Microbiome
Source: F1000Res. 2014 Oct 28;3:257. [Version 1] doi: 10.12688/f1000research.5625.1 (PMC4243741; doi:10.12688/f1000research.5625.1)
Supplement: Peer review history

# Peer review: Referee response for version 1

**DOI:** 10.5256/f1000research.6008.r6563

**Published:** 2014-11-24

**Authors:** Holly M. Brown-Borg

**Affiliations:** 1 Department of Pharmacology, Physiology and Therapeutics, University of North Dakota, Grand Forks, USA

The observations noted in this manuscript partially reflect the subjectiveness of this type of analysis. The results of both manuscripts describe key publications in the field that are skewed unintentionally by search terms.

I have read this submission. I believe that I have an appropriate level of expertise to confirm that it is of an acceptable scientific standard.

# Peer review: Referee response for version 1

**DOI:** 10.5256/f1000research.6008.r6566

**Published:** 2014-11-05

**Authors:** David Lombard

**Affiliations:** 1 Department of Molecular and Cellular Pathology, University of Michigan, Ann Arbor, USA

This piece by Dr. Lamming is informative and concise. The search methodology and results are well described. It should be of interest to researchers in biogerontology.

I have read this submission. I believe that I have an appropriate level of expertise to confirm that it is of an acceptable scientific standard.
